# 771 Enzymatic Burn Wound Debridement: The Learning Curve

**DOI:** 10.1093/jbcr/irad045.246

**Published:** 2023-05-15

**Authors:** Tamara Mertz, Sankhya Sen, Ian Mackie

**Affiliations:** Department of Burns and Plastic Surgery, Southmead Hospital, Bristol, England; Department of Burns and Plastic Surgery, Southmead Hospital, Bristol, England; North Bristol NHS Trust, Bristol, England; Department of Burns and Plastic Surgery, Southmead Hospital, Bristol, England; Department of Burns and Plastic Surgery, Southmead Hospital, Bristol, England; North Bristol NHS Trust, Bristol, England; Department of Burns and Plastic Surgery, Southmead Hospital, Bristol, England; Department of Burns and Plastic Surgery, Southmead Hospital, Bristol, England; North Bristol NHS Trust, Bristol, England

## Abstract

**Introduction:**

The European Medicines Agency (MEA) approved the use of a proteolytic enzymatic debriding agent enriched in bromelain in 2012. It has become a widely used non-surgical treatment alternative for the debridement of deep partial thickness to full thickness thermal burns.

We will present our early experience with this product candidly. We will outline our sequential learning. Moreover, we will describe the pitfalls and lessons learned as well as demonstrate the evolution of our current practice guidelines.

**Methods:**

This is a retrospective analysis of all patients treated with a bromelain based enzymatic debridement agent at a regional burn unit. 17 patients with a mean age of 44.53 years were treated with enzymatic debridement between April 2017 and April 2022. This period includes an 18 month pause where we did not use the product. We reviewed analgesia requirements, efficacy of debridement using the product, wound care post debridement (including the use of skin substitutes), skin grafting post application and overall outcomes.

**Results:**

17 patients were treated during this period. Nine patients had burns involving less than 5% TBSA, three patients had burns between 5 and 15% TBSA and five had burns greater than 15% TBSA.

Of the 17 patients, 10 had the enzymatic debridement agent applied on the ward and seven had it applied in theatre. For the application of the product three patients had general anaesthesia, two had a combination of general and regional anaesthesia (e.g. spinal, axillary block), three had regional analgesia alone, seven received oral analgesia and ward based sedation and two received oral analgesia and ward based Methoxyflurane.

10 patients required skin grafting. One patient was treated with allograft post enzymatic debridement. One patient had their post enzymatic debridement wounds covered with Xenograft. The remainder had their post debridement wounds treated with dressings.

There were three cases where the proteolytic agent failed to debride the burns. In the skin grafted group, 1 patient had complete graft loss.

We will provide a narrative description and outline how each case contributed to the development of our current treatment algorithm.

**Conclusions:**

We now believe that enzymatic debridement does have a real place within the armamentarium of burn wound excision with dermal preservation. Undoubtedly, there is a learning curve associated with its use. This must be considered by any service intending to adopt its use.

**Applicability of Research to Practice:**

We support the use of bromelain enriched proteolytic enzymatic debriding agent in appropriate cases. With experience, it is likely to be useful in the management of many patients who sustain deep partial thickness to full thickness burns.

